# Genomic diversity within the haloalkaliphilic genus *Thioalkalivibrio*

**DOI:** 10.1371/journal.pone.0173517

**Published:** 2017-03-10

**Authors:** Anne-Catherine Ahn, Jan P. Meier-Kolthoff, Lex Overmars, Michael Richter, Tanja Woyke, Dimitry Y. Sorokin, Gerard Muyzer

**Affiliations:** 1 Microbial Systems Ecology, Department of Aquatic Microbiology, Institute for Biodiversity and Ecosystem Dynamics, University of Amsterdam, Amsterdam, The Netherlands; 2 Leibniz Institute DSMZ–German Collection of Microorganisms and Cell Cultures, Braunschweig, Germany; 3 Ribocon, Bremen, Germany; 4 DOE Joint Genome Institute, Walnut Creek, California, United States of America; 5 Winogradsky Institute of Microbiology, Research Centre of Biotechnology, Russian Academy of Sciences, Moscow, Russia; 6 Department of Biotechnology, Delft University of Technology, Delft, The Netherlands; UFRJ, BRAZIL

## Abstract

*Thioalkalivibrio* is a genus of obligate chemolithoautotrophic haloalkaliphilic sulfur-oxidizing bacteria. Their habitat are soda lakes which are dual extreme environments with a pH range from 9.5 to 11 and salt concentrations up to saturation. More than 100 strains of this genus have been isolated from various soda lakes all over the world, but only ten species have been effectively described yet. Therefore, the assignment of the remaining strains to either existing or novel species is important and will further elucidate their genomic diversity as well as give a better general understanding of this genus. Recently, the genomes of 76 *Thioalkalivibrio* strains were sequenced. On these, we applied different methods including (i) 16S rRNA gene sequence analysis, (ii) Multilocus Sequence Analysis (MLSA) based on eight housekeeping genes, (iii) Average Nucleotide Identity based on BLAST (ANI_b_) and MUMmer (ANI_m_), (iv) Tetranucleotide frequency correlation coefficients (TETRA), (v) digital DNA:DNA hybridization (dDDH) as well as (vi) nucleotide- and amino acid-based Genome BLAST Distance Phylogeny (GBDP) analyses. We detected a high genomic diversity by revealing 15 new “genomic” species and 16 new “genomic” subspecies in addition to the ten already described species. Phylogenetic and phylogenomic analyses showed that the genus is not monophyletic, because four strains were clearly separated from the other *Thioalkalivibrio* by type strains from other genera. Therefore, it is recommended to classify the latter group as a novel genus. The biogeographic distribution of *Thioalkalivibrio* suggested that the different “genomic” species can be classified as candidate disjunct or candidate endemic species. This study is a detailed genome-based classification and identification of members within the genus *Thioalkalivibrio*. However, future phenotypical and chemotaxonomical studies will be needed for a full species description of this genus.

## Introduction

Members of the genus *Thioalkalivibrio* are sulfur-oxidizing bacteria that thrive under the dual extreme conditions of soda lakes [1,2]. These lakes are characterized by extremely high sodium carbonate concentrations, creating buffered haloalkaline conditions with a pH of around 10 [3,4]. Despite these extreme conditions, the primary production [5–7] and the microbial diversity [8–11] in these soda lakes is high, and they also contain microbial communities that are actively involved in the cycling of the chemical elements, such as carbon, nitrogen and sulfur [12,13]. Until now, ten species have been validly described within the genus *Thioalkalivibrio* [14–20] and more than 100 strains have been isolated and assigned to this genus [20,21]. The genus *Thioalkalivibrio* is grouped within the gammaproteobacterial family *Ectothiorhodospiraceae* [14]. In addition to their haloalkaliphilic and chemolithoautotrophic nature, the members of this genus are also characterized by a versatile energy metabolism as they are able to use different electron donors and acceptors. All strains can use reduced sulfur compounds, such as sulfide, polysulfide, thiosulfate, polythionates and elemental sulfur as an energy source [14–20]. In addition, the type strains *Tv*. *paradoxus* ARh1^T^ [15], *Tv*. *thiocyanoxidans* ARh2^T^ [15] and *Tv*. *thiocyanodenitrificans* ARhD1^T^ [19] are able to use thiocyanate as their energy, sulfur and nitrogen source [22]. Other type strains, such as *Tv*. *denitrificans* ALJD^T^ [23], *Tv*. *nitratireducens* ALEN2^T^ [17] and *Tv*. *thiocyanodenitrificans* ARhD1^T^ [19] can perform sulfur-dependent denitrification under anaerobic conditions. Moreover, some of the strains can grow over a broad range of salt concentrations (from 0.2 to 5 M Na^+^), and others can even grow with 3.6 M K^+^ [14–20].

By definition, a bacterial species is described as a collection of strains whose DNA:DNA hybridization (DDH) percentage is at least 70% and whose DNA melting temperature (T_m_) lies within 5°C [24]. Apart from these characteristics, a taxonomic species should also reflect a phenotypic coherence [24]. At a higher taxonomic level, a genus is characterized by uniting the assigned strains in a monophyletic branch of a phylogenetic tree, such as 16S rRNA gene sequence analysis or Multilocus Sequence Analysis (MLSA) [25]. In the “All-Species Living Tree Project”, numerous bacterial genera were revealed to be paraphyletic or polyphyletic, which shows that by far not all bacteria are correctly classified at their genus level [26,27]. Whether or not taxa, and in particular genera, are classified in a coherent way, should be assessed, for instance, using modern, genome-based tools as recently shown for the phylum *Bacteroidetes* [28].

Nowadays, in the genomic era, *in silico*-based methods are becoming more and more common [29]. All new genome sequence-based approaches for species delineation have to be however evaluated according to their correspondence to the traditional DDH [30], which ensures consistency in prokaryotic species delineation across hitherto and novel methods. The Average Nucleotide Identity (ANI) was proposed as an *in silico* replacement for the traditional DDH, because it was shown to correlate well with it [31,32] by delineating species from each other using a threshold value of 94–96% [32]. In addition to the ANI calculation, the program JSpecies [32] also provides the tetranucleotide signature correlation index (TETRA) which is a non-alignment based parameter. Another replacement method, the Genome-to-Genome Distance Calculator (GGDC) [33], infers digital DDH (dDDH) estimates from intergenomic distances [33,34] and was shown to provide the highest correlation [33] to conventional DDH without mimicking its pitfalls [35] The dDDH values are predicted on the established DDH scale, along with confidence intervals (CI) that allow conservative taxonomic decisions [33,34] as well as the delineation of bacterial subspecies [36]. The latest GGDC version 2.1 is based on the optimized Genome BLAST Distance Phylogeny (GBDP) method which was originally devised for the inference of highly resolved whole-genome phylogenetic trees using either nucleotide or amino acid data and including branch support [37]. A routine method for the taxonomic classification of bacteria is the analysis of the 16S rRNA gene sequences [30,38] which is however known to have only limited to even no discriminatory power in many bacterial groups [39]. The MLSA approach, which is based on ubiquitous and single-copy housekeeping genes whose proteins have essential and conserved functions, has also been shown to yield highly resolved phylogenetic trees [40, 41]. However, the exclusive application of single-phased and genome-based approaches does still not replace a full and effective taxonomic species description which includes phenotypical, genotypical and chemotaxonomic analysis [42, 43].

Here we describe the genome-based taxonomic classification and identification of strains within the genus *Thioalkalivibrio* in order to assess its genomic diversity. We applied six different approaches on a dataset of 76 *Thioalkalivibrio* genome sequences, such as (i) 16S rRNA gene sequence analysis, (ii) MLSA on eight housekeeping genes (*atpD*, *clpA*, *dnaJ*, *gyrB*, *rpoD*, *rpoH*, *rpoS* and *secF*), (iii) ANI based on BLAST (ANI_b_) and MUMmer (ANI_m_), (iv) tetranucleotide frequency correlation coefficients (TETRA), (v) dDDH and (vi) nucleotide- and amino acid-based GBDP analyses. We revealed 15 new “genomic” species next to the ten already described species, as well as 16 new “genomic” subspecies. We use the term “genomic” species here as the definition of a group of strains which clustered into the same species based on ANI_b_, ANI_m_, TETRA and dDDH analysis. Furthermore, phylogenetic and -genomic analyses showed that the genus is not monophyletic. Finally, species within the genus *Thioalkalivibrio* revealed to have either a candidate disjunct or a candidate endemic biogeographical distribution. This means that they are suggested as a genomic species that harbors strains which are geographically widely separated from each other or that they are only found in a specific area, respectively [44].

## Materials and methods

### Genomes and gene sequences

#### Sequences of *Thioalkalivibrio*

We analyzed the genomic diversity of 76 *Thioalkalivibrio* strains including ten described type strains (S1 Table). The genome sequences of 73 strains were sequenced and annotated within the Community Science Program of the DOE Joint Genome Institute. In addition to these, we sequenced the genomes of *Tv*. *versutus* AL2^T^, *Tv*. *denitrificans* ALJD^T^ and *Tv*. *halophilus* HL17^T^ in order to include all described type strains of *Thioalkalivibrio* in this study.

To obtain these three additional genome sequences, DNA extraction was performed on pure cultures using the PowerSoil DNA Isolation Kit (MoBio Laboratories Inc. (Carlsbad, USA)) following the standard conditions given by the supplier. Paired-end sequencing using Illumina HiSeq 1000 (Illumina; BaseClear B.V. (Leiden, The Netherlands)) was applied. The library was previously prepared by Illumina genomic Nextera XT library. The Illumina reads size was 50 bp and the yield of all three samples was higher than 600 Mb. Quality trimming and genome assembly was done with the CLC Genomics Workbench *de novo* assembler (version 6.0, CLC bio, Aarhus, Denmark) using default settings. The genome sequences were annotated using the Integrated Microbial Genomes Expert Review (IMG-ER) pipeline [45] and deposited in the IMG database under the project ID’s of 62364 (AL2^T^), 62363 (ALJD^T^) and 62362 (HL17^T^) as well as in the NCBI database under the accession of MVAR00000000 (AL2^T^), MVBK00000000 (ALJD^T^) and MUZR00000000 (HL17^T^).

The genome and gene (*clpA*, *atpD*, *gyrB*, *rpoH*, *secF*, *dnaJ*, *rpoD* and *rpoS*) sequences of *Thioalkalivibrio* sp. K90mix and *Tv*. *sulfidiphilus* HL-EbGr7^T^ were obtained from the NCBI RefSeq database and the 16S rRNA gene sequences of the *Thioalkalivibrio* strains AKL11, AL2^T^, ALEN2^T^, ALJ12^T^, ALJ17, ALJ24, ALJD^T^, ALM2^T^, ALSr1, ARhD1^T^, ARh1^T^, ARh2^T^, ARh4, HL17^T^, HL-EbGr7^T^ and K90mix were extracted from the SILVA database [46]. The other *Thioalkalivibrio* genome and gene (*clpA*, *atpD*, *gyrB*, *rpoH*, *secF*, *dnaJ*, *rpoD*, *rpoS* and 16S rRNA) sequences were taken from JGI IMG database [45].

#### Sequences of related species

To study the monophyly of *Thioalkalivibrio* in the phylogenetic and -genomic trees, we selected the closely related *Thiorhodospira sibirica* A12^T^ (photoautotrophic purple sulfur bacterium), *Ectothiorhodospira haloalkaliphila* ATCC 51935^T^ (photoautotrophic purple sulfur bacterium), *Halorhodospira halophila* SL1^T^ (purple sulfur bacterium), *Alkalilimnicola ehrlichii* MLHE-1^T^ (facultatively autotrophic sulfide-oxidizer) and *Thiohalospira halophila* HL3^T^ (extremely halophilic lithoautotrophic sulfur-oxidizer) (S2 Table).

Their 16S rRNA gene sequences were obtained from the SILVA database and the gene sequences for SL1^T^ (with exception of *rpoH*) and MLHE-1^T^ (with exception of *dnaJ*) came from the NCBI RefSeq database. The genome and the gene sequences (*clpA*, *atpD*, *gyrB*, *rpoH*, *secF*, *dnaJ*, *rpoD* and *rpoS*) of A12^T^, ATCC 51935^T^ and HL3^T^ as well as *rpoH* of SL1^T^ and *dnaJ* of MLHE-1^T^ were acquired from the JGI IMG database.

### 16S rRNA gene sequence analysis

Alignment of 16S rRNA gene sequences of the 76 *Thioalkalivibrio* strains and the members of the five related genera was done by the online SINA alignment service [47]. Subsequently, the aligned sequences were imported into ARB [48] by which an identity matrix was calculated. The tree was built in the software program MEGA (version 6.06; [49]) by manually trimming the aligned sequences, and by using the maximum likelihood algorithm as tree inference with 1000 bootstrap replicates, the Tamura-Nei substitution model and gamma distributed with invariant sites (+G+I) as rates among sites. The phylogenetic tree was rooted using *A*. *ehrlichii* MLHE-1^T^ and *H*. *halophila* SL1^T^. In order to calculate the pairwise and overall mean genetic distances with the Kimura 2-parameter model as well as the number of polymorphic sites, the 16S rRNA gene sequences of *Thioalkalivibrio* were aligned with aligner option MUSCLE [50] within MEGA and the ends were trimmed manually to obtain the same length for all sequences.

### Multilocus sequence analysis

The sequences of the individual housekeeping genes of the 76 *Thioalkalivibrio* strains as well as those of the five strains from other genera were aligned with the software program MUSCLE [50] within MEGA (version 6.06; [49]) and trimmed manually. Subsequently, the alignments of the eight genes were concatenated in the following order: *clpA*, *atpD*, *gyrB*, *rpoH*, *secF*, *dnaJ*, *rpoD* and *rpoS*. Phylogenetic trees of individual genes and of the concatenated sequences were calculated in MEGA using the same parameters and the same rooting as for the 16S rRNA gene sequence analysis. The identity matrix of the concatenated housekeeping genes was calculated in MEGA using a pairwise distance matrix made with the “number of difference” model in which also gaps are included as differences. Both, pairwise and overall mean genetic distance as well as the number of polymorphic sites were calculated in analogy to the 16S rRNA gene sequence analysis.

### Average nucleotide identity and TETRA

ANI_b_, ANI_m_ and TETRA values were calculated based on the 76 *Thioalkalivibrio* genome sequences via the JSpeciesWS online service using the default parameters [51].

The resulting matrices obtained for ANI_b_ and ANI_m_ were converted into dendrograms by the DendroUPGMA webservice ([52]; http://genomes.urv.cat/UPGMA/index.php) using an average-linkage clustering [53]. The dendrograms were drawn with the software program Dendroscope 3 [54].

### Whole-genome sequence-based phylogenomic analysis

For all pairwise combinations among the genome sequences of *Thioalkalivibrio* (76) and the members of the other genera (5), intergenomic distances were calculated using the latest version of the GBDP approach [33,55], the software on which the Genome-to-Genome Distance Calculator web service is based (GGDC 2.1; freely available at http://ggdc.dsmz.de) [33]. The inference of pairwise distances included the calculation of 100 replicate distances, each to assess pseudo-bootstrap support [37]. All distance calculations were conducted under the settings recommended for the comparison of nucleotide data [33]. The GBDP trimming algorithm and the formula *d*_*5*_ were chosen because of their benefits regarding phylogenetic reconstruction [37]. Finally, to evaluate potentially less resolved groupings in the nucleotide-based tree, a second GBDP analysis was conducted based on the more conserved amino acid data and under recommended settings [37], i.e., also using the trimming algorithm and formula *d*_*5*_. Afterwards, both phylogenomic trees were inferred from intergenomic GBDP distance matrices using FastME v2.07 with enabled tree bisection and reconnection (TBR) postprocessing [56] (“initial building method”: balanced; “branch lengths assigned to the topology”: balanced; “type of tree swapping (NNI)”: none) and rooted with *A*. *ehrlichii* MLHE-1^T^ and *H*. *halophila* SL1^T^.

### Digital DDH

Using the GGDC 2.1 web service, intergenomic distances were calculated using GBDP [33, 55], followed by the prediction of dDDH values and their CI, for all pairwise comparisons between the genome sequences of the 76 *Thioalkalivibrio* and the 5 type strains of other genera [33].

### Obtaining novel species and subspecies

Since the affiliation of all 76 strains to known type strains is the only relevant taxonomic criterion to assess the actual number of novel species, a previously introduced type-based clustering approach was used to assess the affiliation of strains to known species [57]. The reasoning is that strains within a, for instance, 70% dDDH radius around a known type strain can be safely attributed to the underlying known species or be considered as a novel species else.

In a first step, the different species delineation thresholds were taken from literature and applied to the corresponding dataset in order to identify the strains belonging to a described type species. Therefore, a 70% dDDH radius (including 67% and 73% dDDH that represent its lower and upper CI boundaries) was used for the dDDH dataset, whereas a 94%, 95% and 96% radius for the ANI_b_ and ANI_m_ datasets was used. The TETRA dataset was analysed in the same manner under the published 0.989% and 0.999% thresholds. Since clustering programs frequently require distance data the ANI_b_, ANI_m_ and TETRA similarity matrices were trivially converted to distances (i.e., subtracting the value from 100% and subsequently dividing it by 100). However, the GGDC's intergenomic distances (on which the dDDH is based) could be directly used as input.

In a second step, the strains that were not found to be affiliated to known species (i.e., representing putative novel species) were *de novo*-clustered under the aforementioned thresholds for species delineation. Here, the clustering optimization program OPTSIL was applied in version 1.5 [58] on the dDDH, ANI_b_, ANI_m_ and TETRA matrices to identify these novel species clusters. The OPTSIL program is a tool for the optimization of threshold-based linkage clustering runs [59]. It is primarily driven by two parameters: *T* and *F*. Strains are considered to be “linked” if the pairwise distance is smaller or equal than the chosen threshold *T*. The *F* parameter defines the fraction of links required among a set of strains before merging them into the same cluster. For example, one can either request that it is already sufficient if at least one distance to a cluster member is a link (single linkage; *F* = 0.0) or that all distances are links (complete linkage; *F* = 1.0) [58]. Here, all OPTSIL clustering runs were done with a linkage fraction value *F* set to 0.5, as previously recommended [36].

In a last step, each strain within each putative novel species cluster was consecutively treated as a new putative type strain and the previously described type-based clustering (step 1) was repeated, respectively. In case two or more newly assigned type strains fell into the same species radius, these were counted as “ambiguities”.

Regarding GGDC's capability to delineate microbial subspecies, a respective distance cutoff of 79% dDDH as described in [36] was used.

## Results

### 16S rRNA gene sequence analysis and MLSA

**Fig 1:**
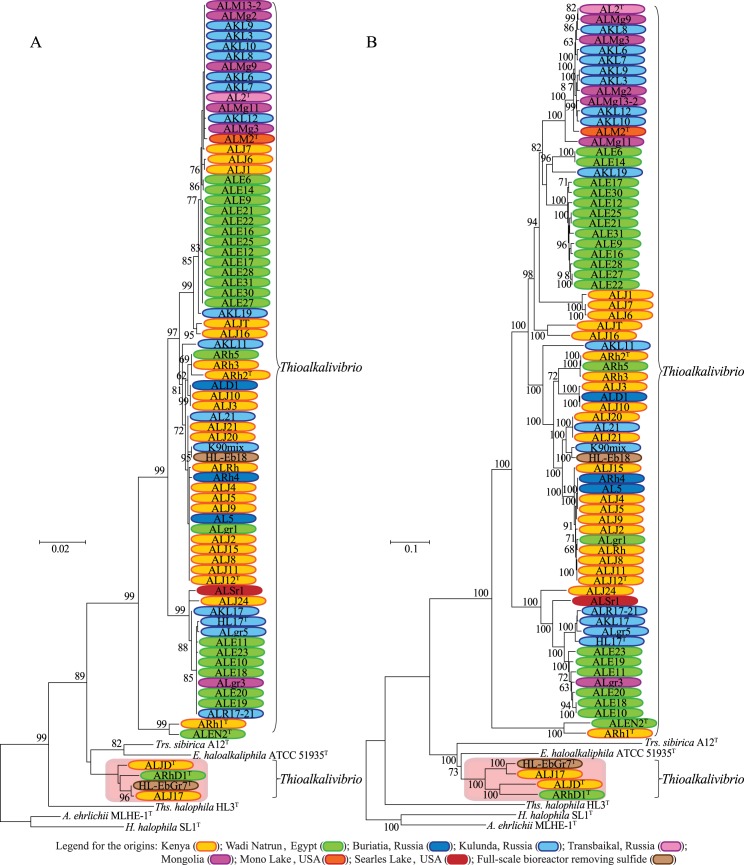
**Phylogenetic tree constructed from 16S rRNA gene sequence analysis (A) and from MLSA (B).** Bootstrap values over 60% were shown at each node. The orange box indicates the outlying *Thioalkalivibrio* strains, contesting the monophyly of the genus.

Phylogenetic trees based on 16S rRNA gene sequences (Fig 1A) and MLSA with eight housekeeping genes (*atpD*, *clpA*, *dnaJ*, *gyrB*, *rpoD*, *rpoH*, *rpoS* and *secF*) (Fig 1B) were constructed for the *Thioalkalivibrio* strains and their close relatives to assess the monophyletic status of the genus.

16S rRNA gene sequence analysis (Fig 1A) and MLSA (Fig 1B) trees showed a separation between the large group of strains around the type species *Tv*. *versutus* AL2^T^ (including the type strains ALM2^T^, ALJ12^T^, ARh2^T^, HL17^T^, ALEN2^T^ and ARh1^T^) and four other *Thioalkalivibrio* strains (ALJD^T^, ARhD1^T^, HL-EbGr7^T^ and ALJ17). This separation was however not well supported in the 16S rRNA tree (bootstrap value of 52%). Two bacteria of different genera, *Trs*. *sibirica* and *E*. *haloalkaliphila*, were situated between the separated groups of the *Thioalkalivibrio* genus (Fig 1).

The alignment of the 16S rRNA gene sequences of the *Thioalkalivibrio* strains has a genetic distance ranging from 0 to 0.0824 (mean 0.0216) which corresponds to a sequence identity from 100 to 92.95% as calculated in ARB (Table 1). These identity results show that the 16S rRNA gene sequence conservation among the different strains of this genus is moderate to high. Especially strains which are closely related, and also some which are classified as different species, possess a relatively high 16S rRNA gene sequence identity value. Furthermore, some nodes in the phylogenetic tree have bootstrap values of less than 60% (Fig 1A).

**Table 1:** Characteristics of 16S rRNA, single housekeeping and concatenated housekeeping genes (MLSA).

Gene	Length (bp)	% of polymorphic sites	Average G+C content (%)	Minimum genetic distance	Maximum genetic distance	Mean genetic distance
16S rRNA	1360	17.94	55.90	0	0.0824	0.0216
*atpD*	1380	34.71	64.32	0	0.1882	0.0895
*clpA*	2299	47.72	64.55	0	0.3221	0.1362
*dnaJ*	1162	51.64	66.92	0	0.3539	0.1521
*gyrB*	2457	54.50	63.11	0	0.4145	0.2000
*rpoD*	1960	46.99	64.24	0	0.2807	0.0934
*rpoH*	897	57.53	66.19	0	0.3988	0.1834
*rpoS*	1156	63.75	64.88	0	0.4402	0.1995
*seqF*	972	55.14	62.16	0	0.4124	0.1987
MLSA	12,283	50.68	64.37	0	0.3179	0.1504

The genetic distance of the MLSA alignment was calculated and ranged from 0 to 0.3179 (mean 0.1504) (Table 1) which corresponds to an MLSA sequence identity from 100 to 75.63% (S4 Table).

The individual single gene trees (S1 File) show only minor differences between each other as well as compared to the MLSA tree (Fig 1B). However, more divergences were found between the MLSA (Fig 1B) and the 16S rRNA gene tree (Fig 1A). On average, MLSA is better resolved and presents longer branches. In the 16S rRNA analysis, the type strain *Tv jannaschii* ALM2^T^ was located on the same branch as the *Tv*. *versutus* AL2^T^ (unsupported though), whereas these type strains were separated on two branches in the MLSA.

### ANI_b_, ANI_m_, TETRA, dDDH and GBDP analyses

**Fig 2:**
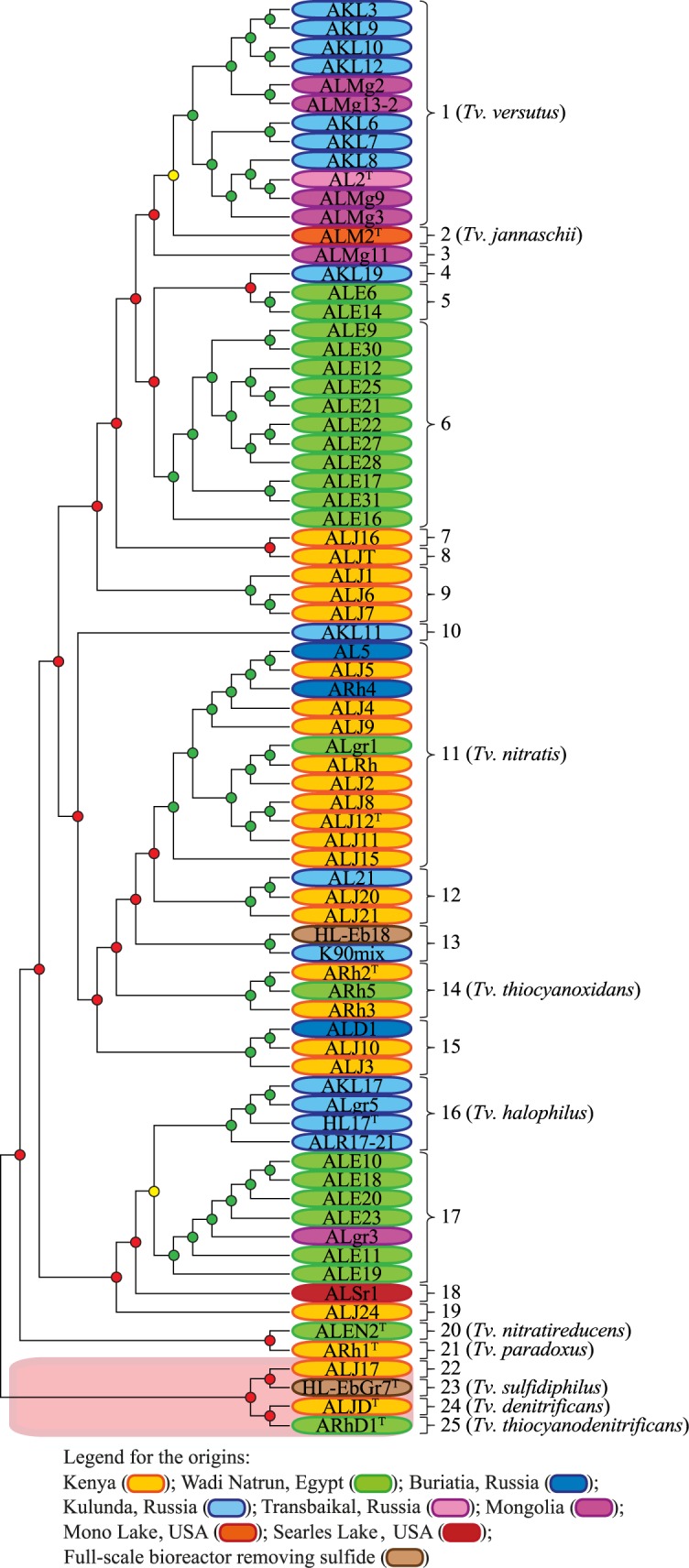
Dendrogram constructed from the ANI_b_ analysis. *De novo* species clusters obtained without consideration of type strains. Clusters are indicated by dots (green: ANI > 96% (strains belong to the same genomic species); yellow: 94% < ANI < 96% (strains might belong to the same genomic species); red: ANI < 94% (strains do not belong to the same genomic species). The genomic species groups are marked by numbers. The orange box indicates the outlying *Thioalkalivibrio* strains, contesting the monophyly of the genus.

**Fig 3:**
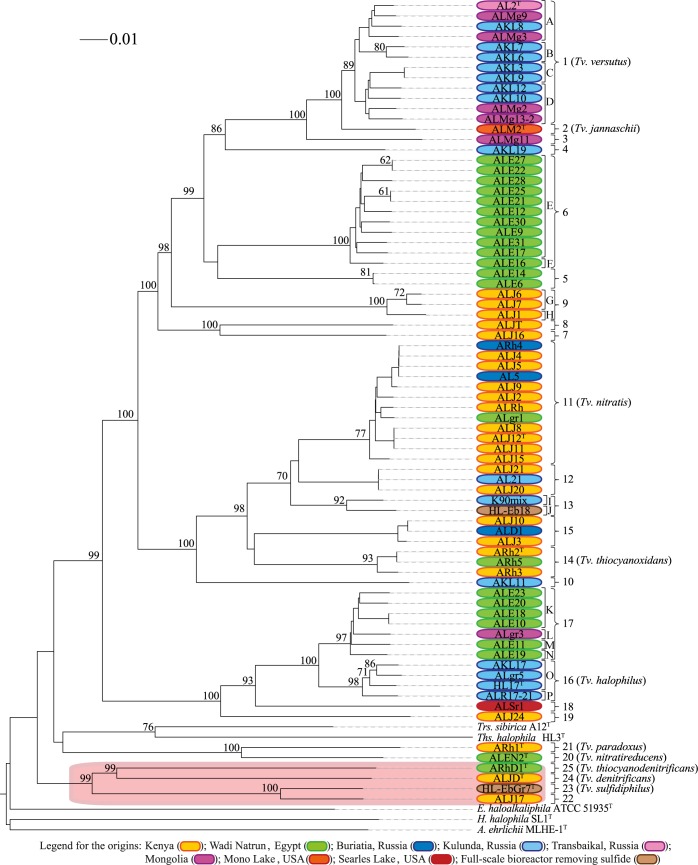
Whole-genome GBDP phylogeny (based on the nucleotide data). Bootstrap values over 60% are shown at each node. An assignment to genomic species was based on the distance threshold equivalent to 70% dDDH (dDDH ≥ 70% indicates same genomic species) and dDDH < 70% (indicates distinct genomic species). Genomic species groups are marked by numbers whereas genomic subspecies groups are denoted by letters. The orange box indicates the outlying *Thioalkalivibrio* strains, contesting the monophyly of the genus.

ANI_b_, ANI_m_, TETRA and dDDH are based on the complete genomic information, enabling the delineation of species among closely-related strains [32,33,35,51]. The ANI_b_ dendrogram is shown in Fig 2. Since dDDH is based on intergenomic GBDP distances, these were used to infer a phylogenomic tree (Fig 3) [37].

The pairwise similarity/distance values for all different measures were calculated and are listed in S5 Table (ANI_b_, ANI_m_, TETRA) and S6 Table (dDDH). The described clustering procedure was applied on all datasets and the resulting clusters are found in S7 Table.

The results of the dDDH dataset (S7 Table) revealed in total 25 non-conflicting (i.e. no ambiguities) genomic species groups under the 70% species delineation threshold, each containing between one and twelve strains per group. From these 25 genomic species groups, 15 new genomic species were identified supplementary to the ten already described species in *Thioalkalivibrio*. The same non-conflicting clusters were also found using the lower CI boundary (67% dDDH). However, the strains AKL3, AKL9 and AKL12 clustered into a group of their own, separated from the other *Tv*. *versutus* strains, under the upper CI boundary (73% dDDH).

Under the 94% delineation threshold, the ANI_b_ dataset (S7 Table) yielded 24 strains that were assigned to multiple type strains (i.e. genomic species groups) at the same time (AL2^T^/ALM2^T^ and HL17^T^/ALE10^PT^) (PT—putative new type strain; chosen to represent its underlying species cluster), whereas, under the 95% threshold delineation threshold, only four of these conflicts were found (AL2^T^/ALM2^T^). At the 96% delineation threshold, the ANI_b_ cluster assignments matched the ones found for the dDDH dataset at the 70% threshold.

The ANI_m_ clustering (S7 Table) revealed 42 strains that fell into multiple species groups under the 94% delineation threshold (AL2^T^/ALM2^T^, ALJ12^T^/HL-Eb18^PT^/AL21^PT^, ALE10^PT^/HL17^T^, ALJ17^PT^/HL-EbGr7^T^ and ALJ12^T^/AL21^PT^), whereas, under the 95% threshold delineation threshold, still 15 strains were ambiguously assigned to multiple genomic species groups (AL2^T^/ALM2^T^ and HL17^T^/ALE10^PT^). At the 96% delineation threshold, the ANI_m_ clustering matched those of the dDDH dataset at the 70% threshold.

TETRA (S7 Table) showed under the 0.989 delineation threshold that almost all strains were ambiguously assigned to multiple genomic species groups at the same time, whereas only 15 strains were affected in that way under the 0.999 delineation threshold (AL2^T^/ALM2^T^/ALMg11^PT^, HL-Eb18^PT^/ALJ12^T^ and ALE10^PT^/HL17^T^).

According to the OPTSIL-based subspecies delineation, using the established dDDH threshold [34], four distinct genomic subspecies were found within the groups 1 (*Tv*. *versutus*) and 17, and two subspecies were identified within the groups 6, 9, 13 and 16 (Fig 3). Trivial subspecies (i.e., a single strain in a given species cluster) were not counted.

**Fig 4:**
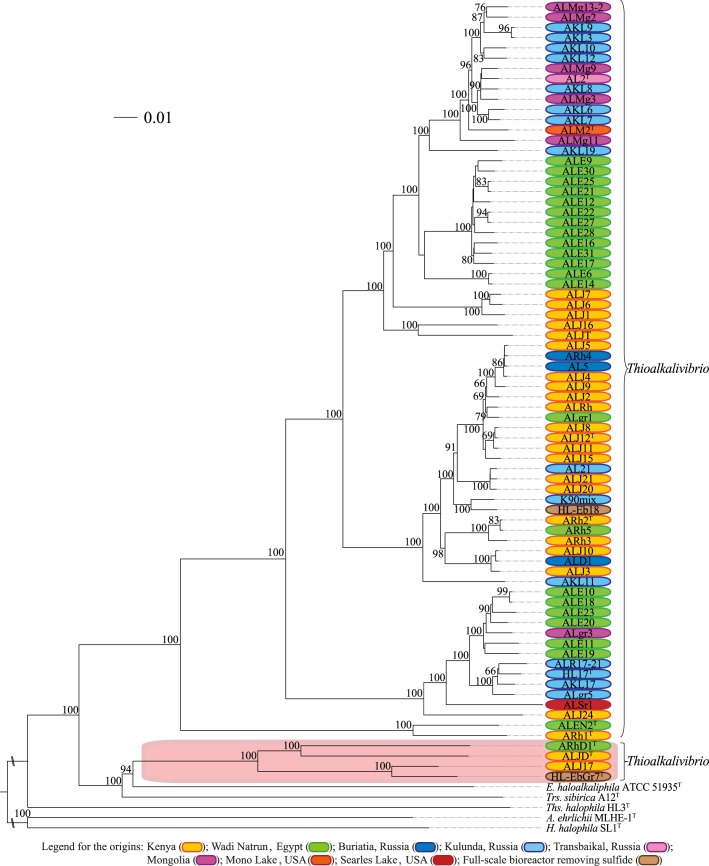
Whole-genome GDBP phylogeny (based on the amino acid data). Bootstrap values over 60% were shown at each node. The orange box indicates the outlying *Thioalkalivibrio* strains, contesting the monophyly of the genus.

Except for the genomic species groups 12 and 15, the nucleotide-based phylogenomic tree (Fig 3) demonstrated that all described type strains could be separated from each other as different genomic species by well supported branches. As expected, on the amino acid-level, the respective phylogenomic tree (Fig 4) revealed even more branch support, including maximum support for the genomic species groups 12 and 15.

Both, the nucleotide- (Fig 3) and the amino acid-based GBDP trees (Fig 4), were inferred to assess the potential monophyly of the genus *Thioalkalivibrio*, which, in fact, turned out to be paraphyletic. In the nucleotide-based tree, in addition to the strains ARhD1^T^, ALJD^T^, HL-EbGr7^T^ and ALJ17, the strains ARh1^T^ and ALEN2^T^ were also separated from the other *Thioalkalivibrio* by *Trs*. *sibirica* and *Ths*. *halophila*. However, neither the relevant subtree of the four strains (ARhD1^T^, ALJD^T^, HL-EbGr7^T^ and ALJ17) nor of ARh1^T^ and ALEN2^T^ was sufficiently supported by this analysis. In the amino acid-based tree, the strains ARhD1^T^, ALJD^T^, HL-EbGr7^T^ and ALJ17 were only separated from the other *Thioalkalivibrio* by *Trs*. *sibirica* and *E*, *haloalkaliphila*, and all relevant nodes yielded high bootstrap values throughout. On average, the nucleotide-based GBDP tree (Fig 3) yielded a bootstrap value of 53.7%, whereas the amino acid-based tree (Fig 4) was generally better resolved with an average support of 81.5%, as expected [37].

## Discussion

### Species classification and identification in *Thioalkalivibrio*

The 76 *Thioalkalivibrio* strains could not be uniformly classified into different sets of species groups by ANI_b_, ANI_m_, TETRA and dDDH. In the dDDH dataset, all strains were non-ambiguously assigned either to one of the known species or they represented new ones (Fig 3 and S7 Table). The clustering based on ANI_b_ and ANI_m_ revealed conflicts at the 94% and 95% thresholds, however gave the same non-ambiguous genomic species clusters at the 96% threshold as the dDDH at 70% (Fig 2, S1 Fig and S7 Table). The TETRA results showed a high number of conflicts under the 0.989 threshold and a few with 0.999 threshold. A possible reason for the non-conflicting results of dDDH might be due to its better correlations to conventional DDH [33], the main optimality criterion for all such *in silico* methods. Even though, clustering inconsistencies of ANI_b_ data were previously observed [60], performance parameters, such as cluster consistency, isolation and cohesion indices [34,36], would need to be investigated for a large, representative dataset of bacteria and archaea, as successfully done earlier for dDDH data [34]. Consequently, it seems to be premature to infer any conclusions regarding the (un-)reliability of the other methods, just based on this study.

Among the 25 genomic species clusters, ten were within the radius of an existing type strain and could thus be successfully linked to a described species. Consequently, the 15 remaining groups did not contain a described type strain and therefore, novel species are proposed to be effectively described within the genus *Thioalkalivibrio* in accordance with the taxonomic rules. These genomic species need to be evaluated by a polyphasic approach in which they need to have a sufficient level of phenotypic and physiological differences with already described species [24,42,43]. The aforementioned clustering conflicts should be carefully investigated in the course of these effective species descriptions, because they might reflect a phenotypic coherence [24].

Furthermore, multiple subspecies groups were found within the genomic species groups 1 (*Tv*. *versutus*), 6, 9, 13, 16 and 17 (Fig 3) using the GBDP nucleotide-based analysis [36]. Even though an assignment to subspecies is usually only done for medically relevant strains, we used this approach to gain a better understanding about the diversity within the genus *Thioalkalivibrio*.

A high genomic diversity is reflected in *Thioalkalivibrio* through the large number of discovered genomic species and subspecies affiliated to *Thioalkalivibrio*. Branching patterns of rep-PCR profiles of *Thioalkalivibrio* strains might indicate that the diversity in *Thioalkalivibrio* originates from recombination [61]. It is already known that recombination plays an important role in the evolution and diversification of bacterial species [62–64], even more so than mutations [65,66]. Multiple transposases have already been found in the genome of *Thioalkalivibrio* sp. K90mix [67] and pathogenicity islands as well as prophages in *Tv*. *versutus* D301 [68]. Further studies will aid in the clarification of the nature and proportions of the evolutional forces responsible for the diversification within the genus *Thioalkalivibrio*.

In this study, we found that various *Thioalkalivibrio* strains have previously been misidentified (S8 Table) [14,20]. Furthermore, the previous studies [69,70,71] consider the strain ALJ15 to represent *Tv*. *versutus*, which we identified as a member of the species *Tv*. *nitratis*.

16S rRNA gene sequence analysis yielded high identity values among closely related strains and species, and the phylogeny was not well supported. For this reason, this analysis can only distinguish between different *Thioalkalivibrio* species at a low resolution, which was previously observed for other bacteria [72,39], such as *Hyphomonas* [73], *Thalassospira* [74], *Acinetobacter* [75], *Nocardia* [76] and *Bifidobacterium* [77]. Therefore, species affiliation cannot be based on 16S rRNA gene sequence analysis alone due to the fact that different taxa might have different diversification rates of their 16S rRNA gene sequences [78]. Additionally, incorrect assignments can be made using only a single housekeeping gene such as the 16S rRNA gene sequence, because horizontal gene transfer might even occur (though unlikely) for the 16S rRNA gene sequence [79–81]. Indeed, different studies demonstrated that a higher taxonomic resolution and consistency in accepted classification is achieved using a set of at least five housekeeping genes in MLSA [29,36,82,83] or in supertree analysis with single-copy orthologous core genes [75]. It was even demonstrated that the taxonomy of whole phyla can be extensively and reliably revised based on the principles of phylogenetic classification and trees inferred from genome-scale data [28]. In this study, the GBDP (Figs 3 and 4) and MLSA (Fig 1B) showed on average a better resolution, higher bootstrap values and more clusters than the 16S rRNA gene sequence analysis (Fig 1A), supporting the expected higher distinguishing power of these methods.

Comparing the identity results of the MLSA to those of the ANI_b_ and the values of the dDDH, a threshold value for the genomic species delimitation based on the sequence identity given by MLSA could be proposed (S4 Table). With the set of strains and gene sequences used in this study, it was found that strains with a sequence identity higher than 98.13% belong to the same genomic species, whereas identity values below 97.77% indicated that they were not associated to the same genomic species. In between these two values, a grey area exists. However, these values might change if new strains are added in the future to the current set of strains. With this knowledge, we propose that MLSA can be used as a fast and preliminary assessment of the species relatedness for new isolates in *Thioalkalivibrio*. This method has the advantage that the whole genome sequence is not needed (at this point) and it provides more phylogenetic resolution at species level than the 16S rRNA gene sequence analysis for *Thioalkalivibrio*. However, the 16S rRNA gene sequence still has the advantage of having a large database linked to it. If genome sequences are available, respective whole-genome sequence-based approaches should be preferred and chosen regarding their clustering performance assessed in this comprehensive study.

### *Thioalkalivibrio*’s phyletic structure at genus level

The genus *Thioalkalivibrio* is not monophyletic according to the phylogenetic and phylogenomic analyses (Figs 1, 3 and 4), because type strains from other genera disconnect a group of strains including *Tv*. *sulfidiphilus* HL-EbGr7^T^, ALJ17, *Tv*. *denitrificans* ALJD^T^ and *Tv*. *thiocyanodenitrificans* ARhD1^T^ from the major group of *Thioalkalivibrio* that includes their type species *Tv*. *versutus*. The amino acid-based GBDP analysis supported the MLSA in this respect and, furthermore, yielded higher bootstrap values for all relevant nodes. This is explained by the more conserved nature of the amino acid sequences as well as that GBDP is bootstrapping entire genes [37] which was previously suggested to reduce conflicts and to provide more realistic support values in phylogenomic analyses [28,84]. The 16S rRNA gene sequence showed the same separation as found in the MLSA and the nucleotide-based GBDP, but this node achieved only low branch support. The nucleotide-based GBDP analysis showed that in addition to the strains which were separated in the MLSA and amino acid-based GBDP (ARhD1^T^, ALJD^T^, HL-EbGr7^T^ and ALJ17), the strains ARh1^T^ and ALEN2^T^ were also separated from the other *Thioalkalivibrio*. However, neither the relevant subtree of the four strains (ARhD1^T^, ALJD^T^, HL-EbGr7^T^ and ALJ17) nor of ARh1^T^ and ALEN2^T^ was sufficiently supported in this analysis.

In the 16S rRNA gene sequence analysis, the MLSA and the amino acid-based GBDP, the genus *Thioalkalivibrio* is split into two groups by *Trs*. *sibirica* and *E*. *haloalkaliphila*. However, in the nucleotide-based GBDP, *Ths*. *halophila* is found instead of *E*. *haloalkaliphila* in between the two *Thioalkalivibrio* groups. The bacteria *Trs*. *sibirica* and *E*. *haloalkaliphila* are both anaerobic and haloalkaliphilic purple sulfur bacteria isolated from soda lakes [85,86]. However, due to the fact that *Trs*. *sibirica* and *E*. *haloalkaliphila* have a different energy metabolism [85,86], they do not adhere to the description of the *Thioalkalivibrio* genus, which is obligatory chemotrophic [87]. *Ths*. *halophila* is a chemolithoautotrophic and haloneutrophilic sulfur oxidizing bacterium which originates from hypersaline inland lakes. Furthermore, the *Thiohalospira* genus also contains the facultatively alkaliphilic species *Ths*. *alkaliphila* [88]. Physiologically, the four separated *Thioalkalivibrio* strains are closer to the *Thiohalospira* genus with the exception of their alkaliphilic nature [14,19,20,88].

A taxonomic genus must be monophyletic by definition [25,89]. In the case of a monophyletic group, all members share a common ancestor and therefore, it is possible to detach the group from the tree with a single cut [90]. For this reason, the four strains (HL-EbGr7^T^, ALJ17, ALJD^T^, ARhD1^T^) of *Thioalkalivibrio* which are separated from the major group of *Thioalkalivibrio* that contain the type strain *Tv*. *versutus* AL2^T^, cannot remain within the same genus and need to be reclassified into a new genus. However, no fixed and commonly accepted boundary for genus delineation exists, which could be used to clarify the genus boundary in *Thioalkalivibrio*. This is a known circumstance in microbial taxonomy which is primarily due to the missing ultrametricity [34] in such biological data, especially regarding ranks above species level. In the “All-Species Living Tree Project”, a minimal identity value of the 16S rRNA gene sequence for the separation of two genera was proposed at 94.8% ± 0.25 [91]. Applying this value to the 16S rRNA gene sequence analysis of *Thioalkalivibrio* (S3 Table), the splitting of the two groups in the phylogenetic tree was confirmed (92.95–94.92%; mean = 93.82%) (S3 Table). Furthermore, the identity values between the four outliers (HL-EbGr7^T^, ALJ17, ALJD^T^, ARhD1^T^) and *Ths*. *alkaliphila* are also below this value (91.86–92.22%) (S3 Table). Other findings from the “All-Species Living Tree Project” demonstrate that several genera as *Eubacterium*, *Bacillus*, *Pseudomonas*, *Desulfotomaculum* [26], *Enterococcus*, *Rhizobium*, *Clostridium* and *Lactobacillus* [27] are paraphyletic or polyphyletic. These examples indeed visualize that misclassifications are not an uncommon problem, especially when species descriptions were ultimately based on unresolved, hence uninterpretable, 16S rRNA gene sequence trees.

On the basis of their phenotypic characteristics, the outliers also showed differences to the core group of *Thioalkalivibrio*. The ability of growing at higher salinity ranges of up to 5 M of Na^+^ is linked to many genomic species in the core group containing the type species, *Tv*. *versutus*, whereas the type strains *Tv*. *nitratireducens* ALEN2^T^, *Tv*. *paradoxus* ARh1^T^, *Tv*. *sulfidiphilus* HL-EbGr7^T^, *Tv*. *denitrificans* ALJD^T^ and *Tv*. *thiocyanodenitrificans* ARhD1^T^ which are genetically further away from their type species, do not have an adaptation to high salt concentrations [14–20].

### Biogeography

Given the currently available *Thioalkalivibrio* sequences, we were able to infer a relation between the geographic origin and the genomic relatedness of the strains with the results of this study (Figs 1–4, S1 Fig). The strains were isolated from soda lakes including Kenya (24 strains), Egypt (23 strains), Buriatia (Russia)(3 strains), Kulunda Steppe (Altai, Russia)(15 strains), Transbaikal region (Russia)(1 strains), North-eastern Mongolia (6 strains), Mono and Searles Lakes in California (USA)(2 strains), as well as from a haloalkaline H_2_S-removing bioreactor (2 strains).

Based on the set of genome sequences used in this study, some genomic species groups might be suggested to have a candidate endemic biogeographic distribution [44], such as the genomic species group 1 (*Tv*. *versutus*), which has so far only been isolated from Central Asian soda lakes, group 16 (*Tv*. *halophilus*), which comes from south-western Siberia, as well as the genomic species groups 5 (Egypt), 6 (Egypt) and 9 (Kenya). Other genomic species contain strains that are geographically widely separated from each other. Therefore, it was suggested to classify those in a candidate disjunct distribution [44]. The genomic species groups 11 (*Tv*. *nitratis*), 14 (*Tv*. *thiocyanoxidans*) and 17 are primarily found in one area, but also included isolates from other distant locations. Different isolation locations are also observed in the genomic species groups 12, 13, 14, 15 and 17, which contain only two or three strains, and therefore, no statement regarding their dispersion can be made. Nevertheless, using our dataset, it can generally be concluded that most genomic species tend to occur in one geographical region such as Central Asia (Mongolia and south Siberian steppes), Kenya or Egypt. The preference for specific locations might correspond to a better adaptation to certain local environmental conditions. Obvious characteristics distinguishing the different locations might be the fluctuations in temperature and the incoming freshwater during the year, as well as the ratio between sodium carbonate and sodium chloride. In particular, the Central Asian soda lakes are characterized by hot summer, freezing winter and a significant brine dilution due to snow melting in spring time. The Wadi Natrun and Searles lakes are characterized by a domination of chlorides over carbonates.

Several studies reported endemicity in different bacterial groups including *Hyphomonas* [73], *Tenacibaculum* [92], fluorescent *Pseudomonas* strains [93], 3-chlorobenzoate-degrading soil bacteria [94], hot spring cyanobacteria [95] and the hyperthermophilic Archaea *Sulfolobus* [96,97]. [61] studied the genomic diversity and the biogeography by means of rep-PCR and found that most genotypes were bound to a specific region for which an endemic distribution was suggested. However in our results, a disjunct distribution is seen for most *Thioalkalivibrio* species. It is important to note that only 29 strains were in common in both analyses and thus, a different picture of the geographical dispersion can be produced. Comparing the clustering of the strains common in both studies, the same structure was generally observed. However, some differences are still present as for example the splitting of the genomic species groups 1 (*Tv*. *versutus*) and 11 (*Tv*. *nitratis*) in the clustering constructed by the rep-PCR profile. Thus, until now, these results provide no clear conclusion on the biogeography of the *Thioalkalivibrio* genus yet.

Soda lakes are remotely located extreme habitats. To allow migration and dispersion of *Thioalkalivibrio* in between the different lakes, bird migration or transportation by particles of sand, salt or dust might be used [61]. For these journeys, they need to be equipped against drought and starvation by forming a resting cell form, called cyst-like refractile cells [98], as well as by producing a yellow pigmentation protecting against UV light [71], high salinity and oxidative stress [70]. However, these types of transportation are likely limited to locations in each area and between the African and Asian continent, while the American continent is further isolated from the African and Asian isolation sites. Nevertheless, *Tv*. *jannaschii* ALM2^T^ isolated from Mono Lake (USA) presents high genomic relatedness to *Tv*. *versutus* AL2^T^ isolated from Transbaikal region (Russia), which might be due to a recent separation or a change in the advance of the molecular clock.

However, to obtain a broader and a more robust view on the species dispersion at a worldwide scale and on a possibly endemic, disjunct or cosmopolitan distribution, the number of studied strains should be considerably increased for example by using metagenomic datasets and their origins should be chosen more homogeneously on a world-wide scale.

## Conclusions

The genus *Thioalkalivibrio* is more diverse at its species and subspecies level than known before. We discovered 15 novel genomic species and 16 genomic subspecies in addition to the ten already described species. Furthermore, the non-described strains were successfully classified into the different genomic species. The analyses also revealed that *Thioalkalivibrio* is not a monophyletic genus, because other genera of haloalkaliphilic sulfur bacteria clearly separate four *Thioalkalivibrio* strains from the core group clustering around the type species *Tv*. *versutus* AL2^T^. Therefore, these four outliers need to be split from the current genus and to be reclassified into a new genus. Furthermore, the different genomic species can either be classified as candidate disjunct or candidate endemic. In this study, we provide a backbone for the genomic classification of currently available *Thioalkalivibrio* strains, as well as for new strains. In the future, the here proposed new species should be effectively described according to current taxonomic conventions via a polyphasic approach.

## Supporting information

S1 Fig Dendrogram based on ANI_m_. *De novo* species clusters obtained without consideration of type strains. Clusters are indicated by dots (green: ANI > 96% (strains belong to the same genomic species); yellow: 94% < ANI < 96% (strains might belong to the same genomic species); red: ANI < 94% (strains do not belong to the same genomic species). The origin of the strains is indicated with different colors (see legend of Fig 1).
(PDF)

S1 Table Genome characteristics of *Thioalkalivibrio* strains used in this study. (DOCX)

S2 Table Genome characteristics of the other genera used in this study. (DOCX)

S3 Table 16S rRNA gene sequence identities. (XLSX)

S4 Table Identity values based on MLSA. (XLSX)

S5 Table Calculated ANI_b_, ANI_m_ and TETRA values. Strains marked with a (T) are type strains. Genomic species classification based on ANI_b_ and ANI_m_ value (green: ANI > 96% (strains belong to the same genomic species); yellow: 94% < ANI < 96% (strains might belong to the same genomic species); black: ANI < 94% (strains do not belong to the same genomic species). Genomic species classification based on TETRA value (green: TETRA > 0.999% (strains belong to the same genomic species); yellow: 0.989% < TETRA < 0.999% (strains might belong to the same genomic species); black: TETRA < 0.989% (strains do not belong to the same genomic species).
(XLSX)

S6 Table Predicted dDDH values. Strains marked with a (T) are type strains. Genomic species classification based on dDDH shown by dots (green: dDDH ≥ 70% (strains belong to the same genomic species); black: dDDH < 70% (strains do not belong to the same genomic species).
(XLSX)

S7 Table OPTSIL *de novo* species clustering and affiliation, and type-based affiliation results of dDDH, ANI_b_, ANI_m_ and TETRA. (XLSX)

S8 Table Previous and current species affiliations. (XLSX)

S9 Table Nucleotide- and amino acid-based GBDP distance matrices. (XLSX)

S1 File Single gene phylogenetic trees based on *atpD*, *clpA*, *dnaJ*, *gyrB*, *rpoD*, *rpoH*, *rpoS* and *secF* gene sequences. (PDF)
