# Supplementary figures and images for: Genomic diversity within the haloalkaliphilic genus Thioalkalivibrio
Source: PLoS One. 2017 Mar 10;12(3):e0173517. doi: 10.1371/journal.pone.0173517 (PMC5345834; doi:10.1371/journal.pone.0173517)

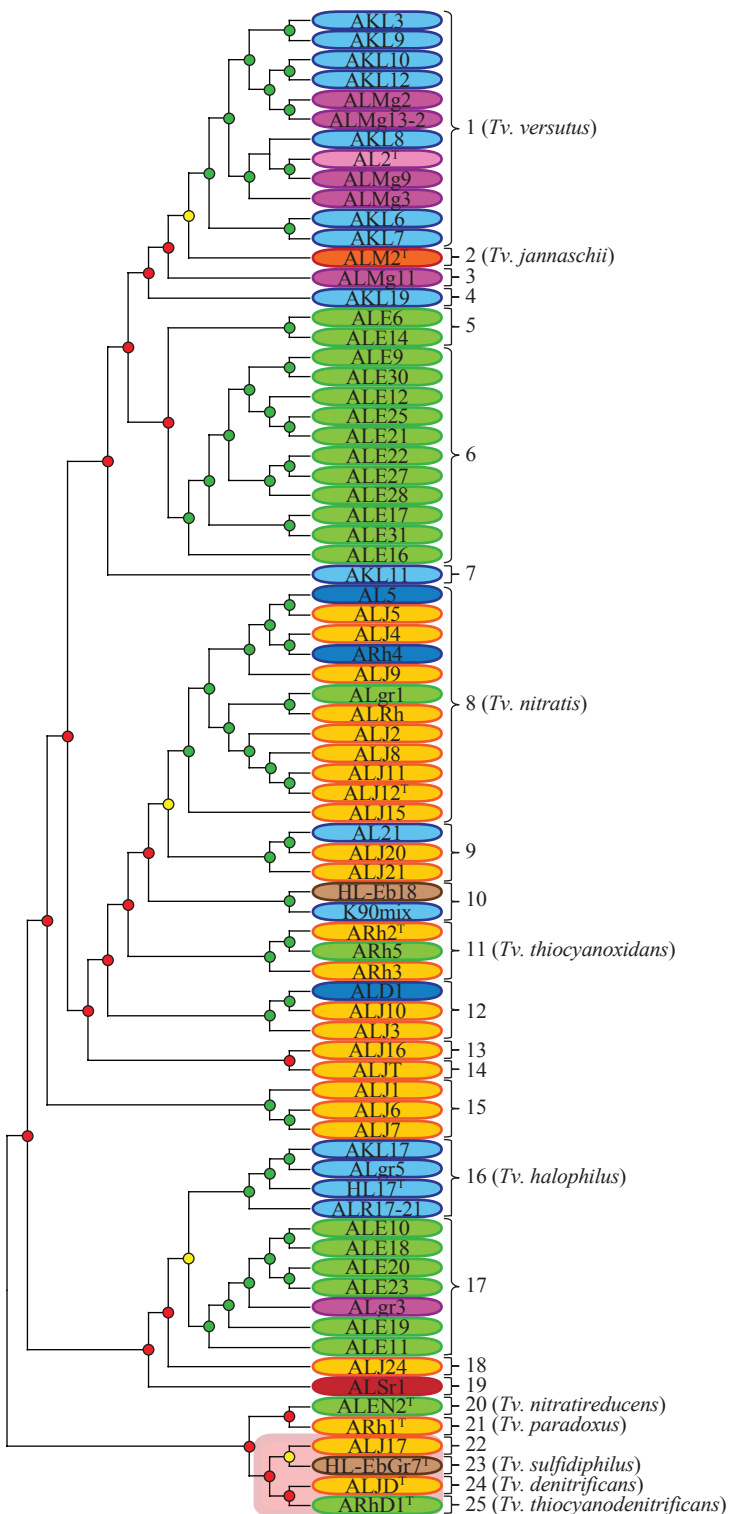

Supplement: S1 Fig — De novo species clusters obtained without consideration of type strains. Clusters are indicated by dots (green: ANI > 96% (strains belong to the same genomic species); yellow: 94% < ANI < 96% (strains might belong to the same genomic species); red: ANI < 94% (strains do not belong to the same genomic species). The origin of the strains is indicated with different colors (see legend of Fig 1). (PDF) [file pone.0173517.s001.pdf]

*atpD*

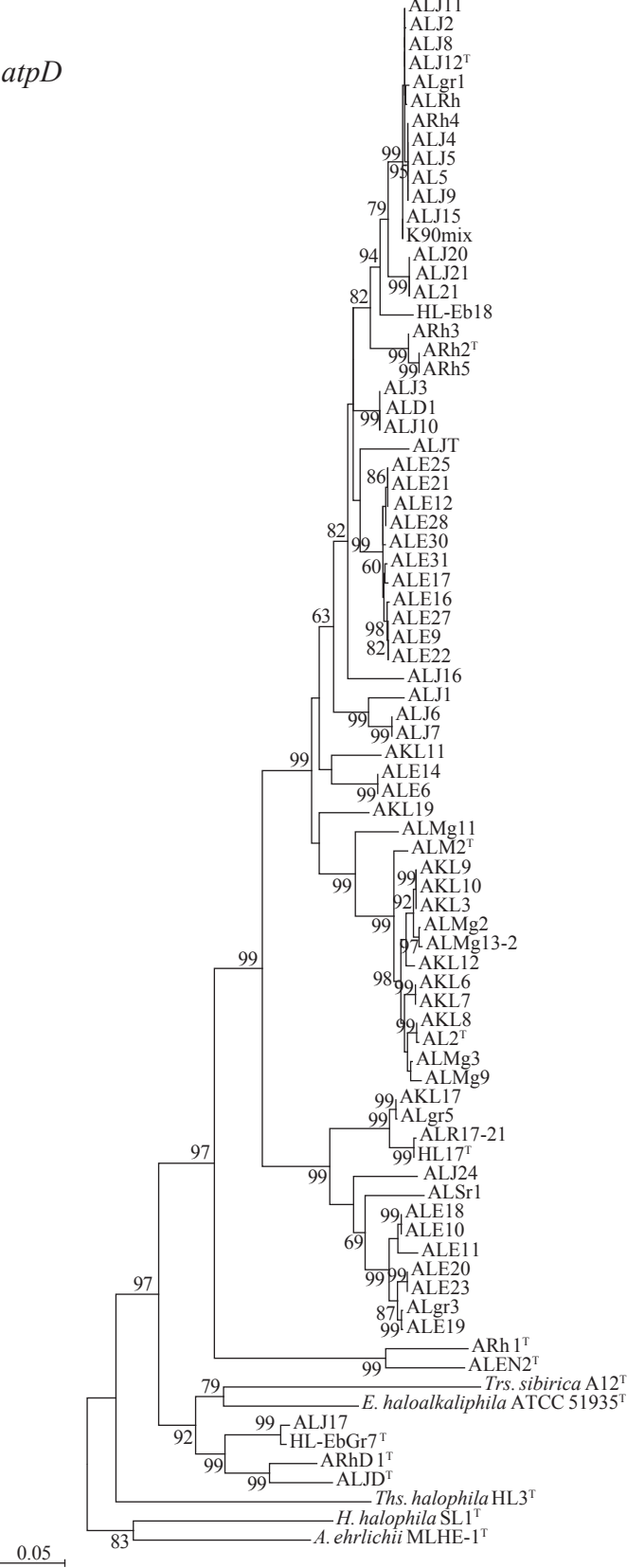

*clpA*

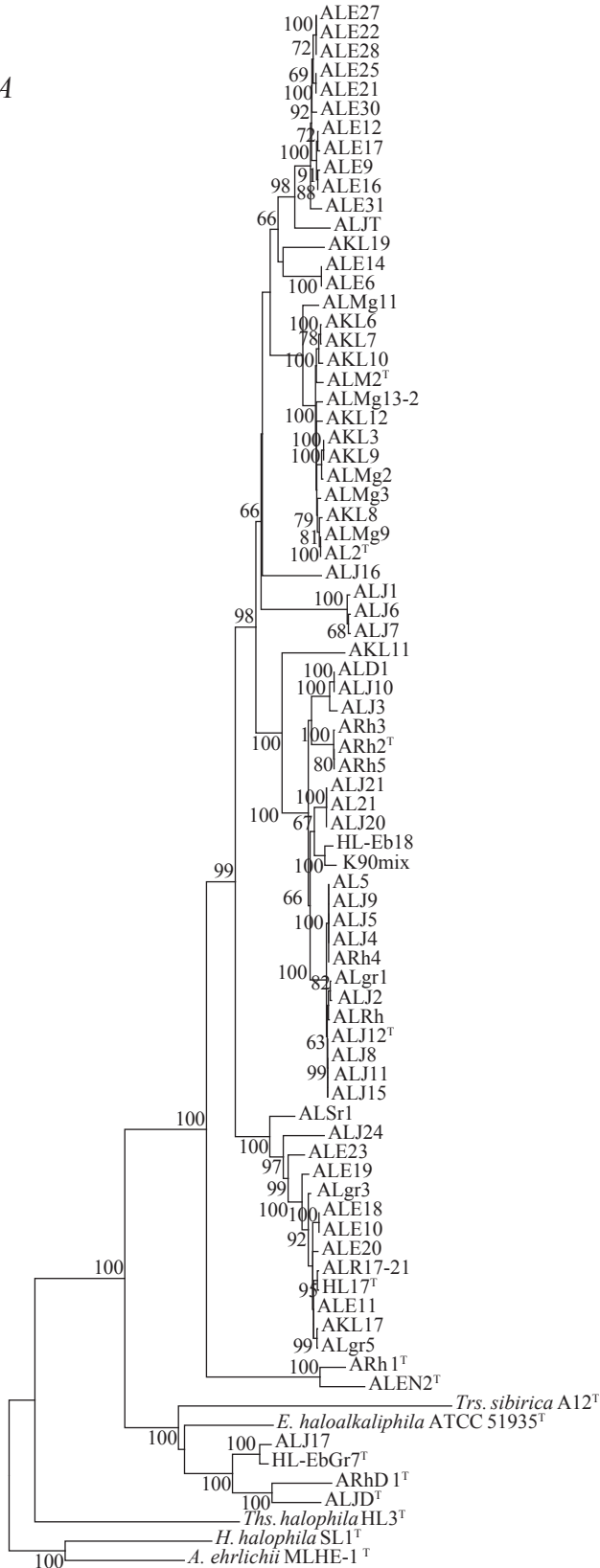

0.05

*dnaJ*

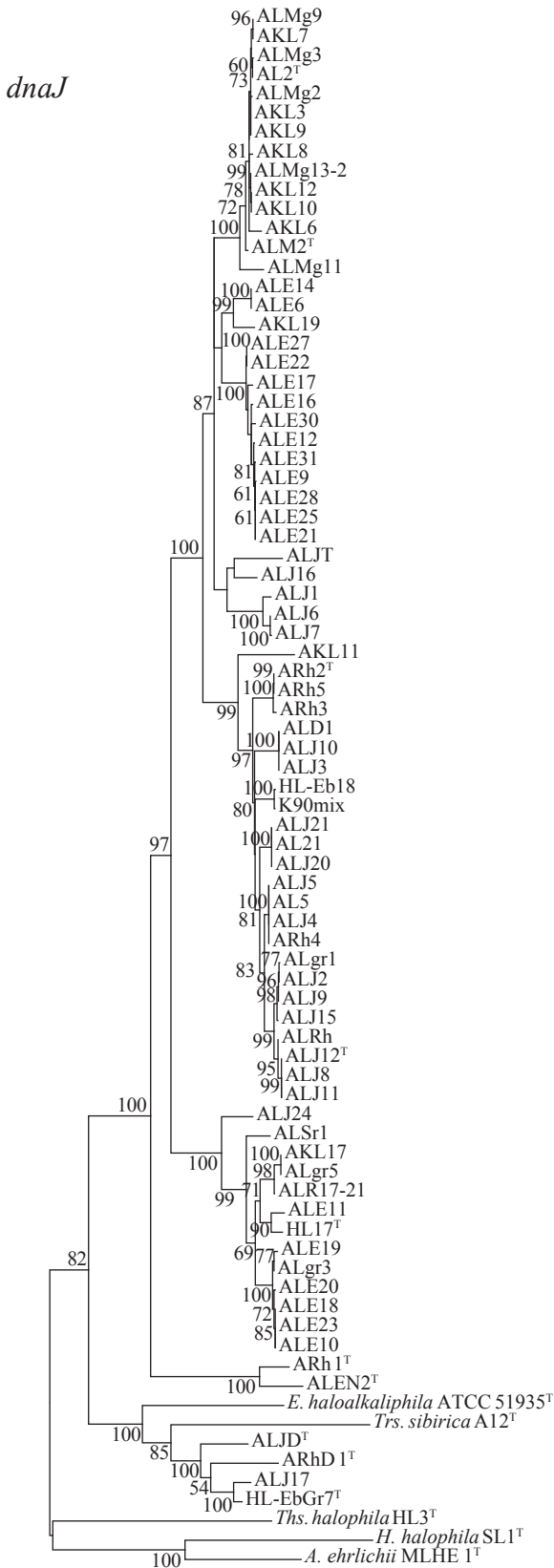

*gyrB*

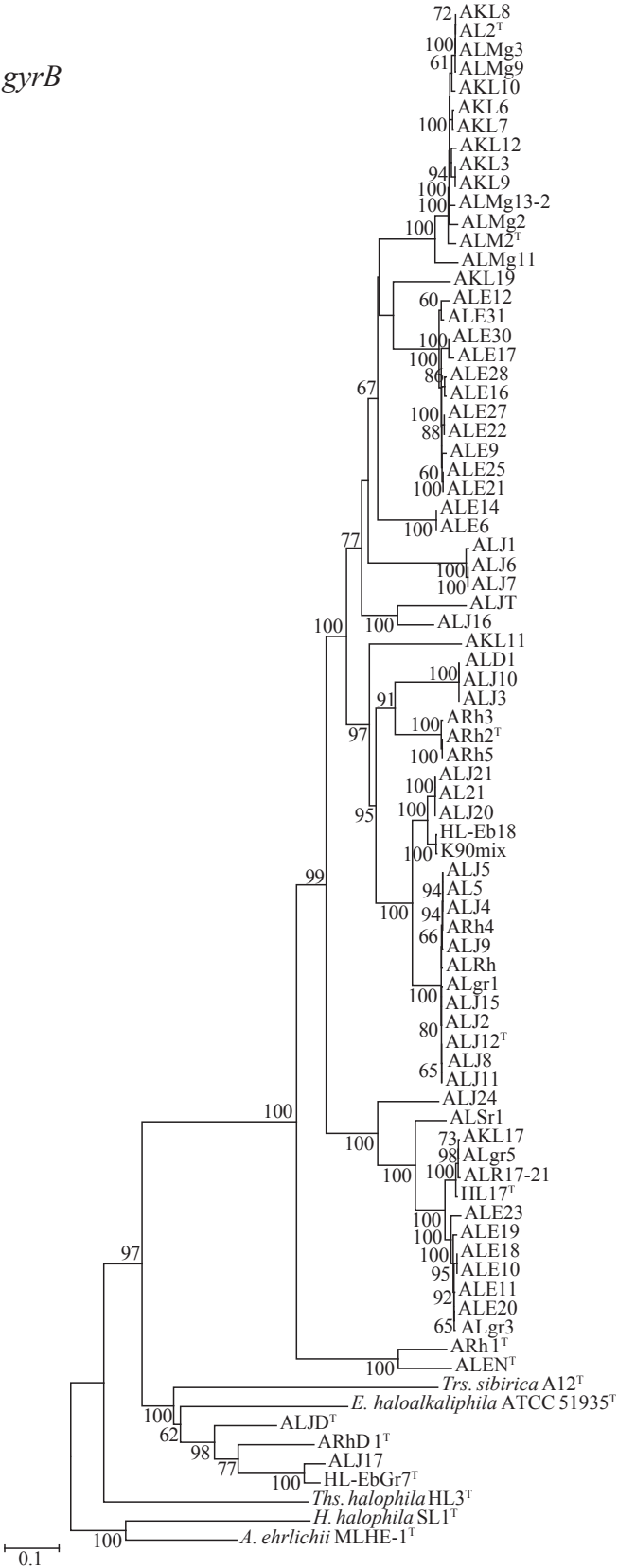

*rpoD*

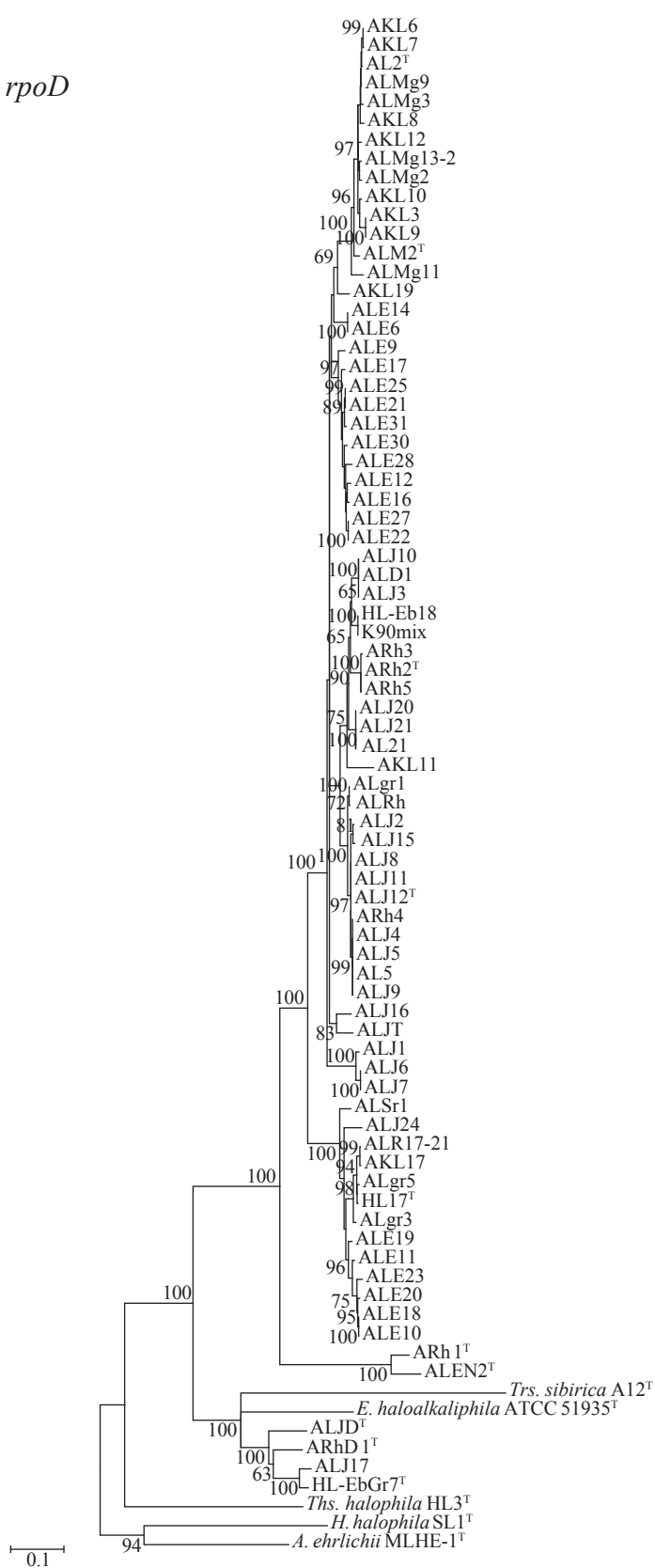

*rpoH*

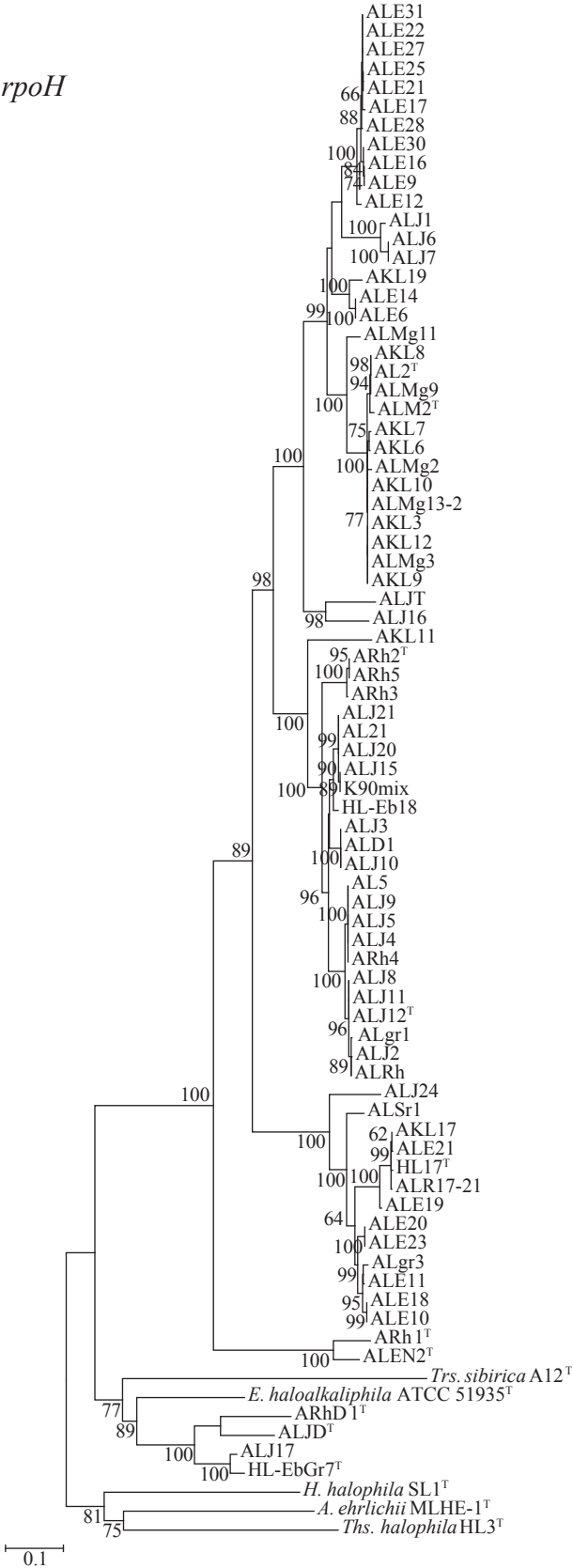

*rpoS*

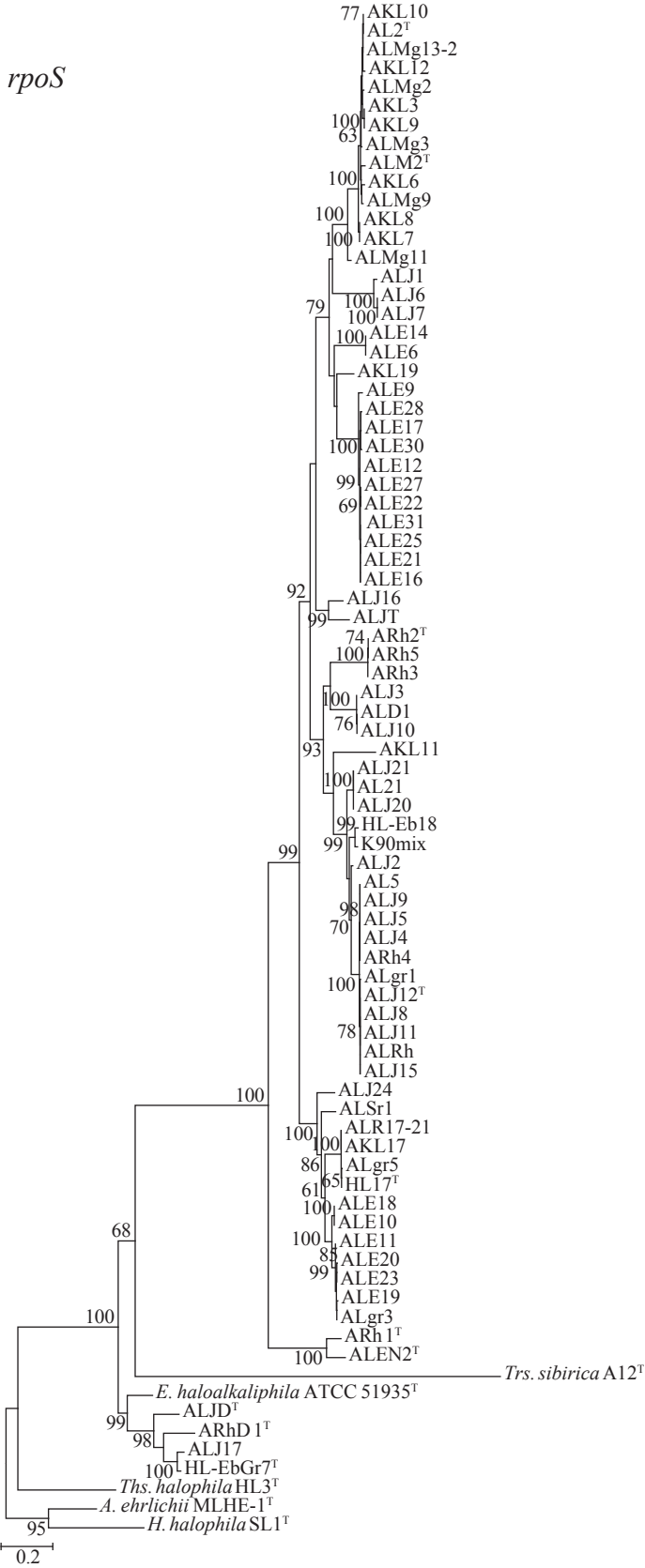

*secF*

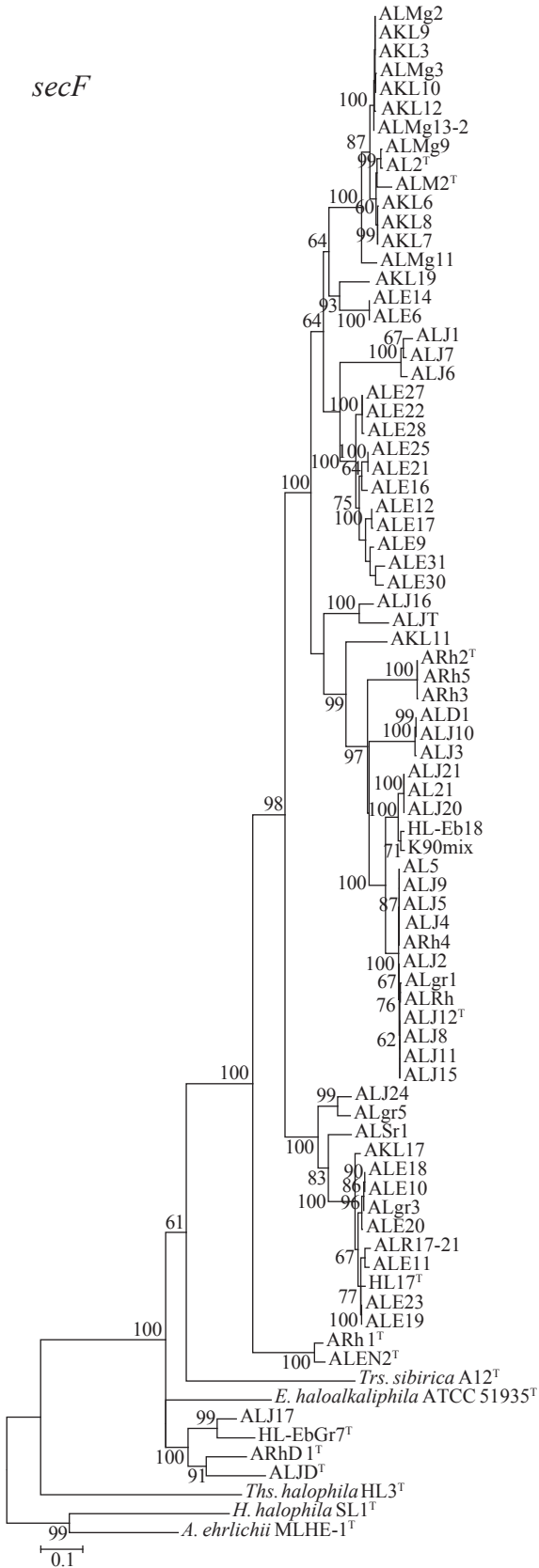

Supplement: S1 File — (PDF) [file pone.0173517.s011.pdf]
