# Supplementary material for: Genomic diversity within the haloalkaliphilic genus Thioalkalivibrio
Source: PLoS One. 2017 Mar 10;12(3):e0173517. doi: 10.1371/journal.pone.0173517 (PMC5345834; doi:10.1371/journal.pone.0173517)
Supplement: S1 Table — (DOCX) [file pone.0173517.s002.docx]

S1 Table. Genome characteristics of *Thioalkalivibrio* strains used in this study.

| **Strain name** | **GOLD ID** | **Genbank assembly accession** | **Location of isolation** | **Genome size (bp)** | **No. of scaffolds** | **Scaffold N50** | **G/C ratio (%)** | **Predicted CDS** |
| --- | --- | --- | --- | --- | --- | --- | --- | --- |
| AKL3 | Ga0000126 | GCA_000377805.1 | Kulunda Steppe, Altai, Russia | 2,820,283 | 33 | 136,329 | 66.26 | 2743 |
| AKL6 | Ga0000127 | GCA_000376905.1 | Kulunda Steppe, Altai, Russia | 2,836,380 | 11 | 575,221 | 66.42 | 2707 |
| AKL7 | Ga0000128 | GCA_000381705.1 | Kulunda Steppe, Altai, Russia | 2,874,105 | 20 | 418,649 | 66.32 | 2748 |
| AKL8 | Ga0000129 | GCA_000380525.1 | Kulunda Steppe, Altai, Russia | 2,842,874 | 14 | 328,285 | 66.44 | 2711 |
| AKL9 | Ga0000130 | GCA_000377825.1 | Kulunda Steppe, Altai, Russia | 2,822,887 | 29 | 194,940 | 66.26 | 2744 |
| AKL10 | Ga0000122 | GCA_000381845.1 | Kulunda Steppe, Altai, Russia | 2,986,682 | 8 | 636,200 | 66.1 | 2848 |
| AKL11 | Ga0000123 | GCA_000377845.1 | Kulunda Steppe, Altai, Russia | 3,002,444 | 20 | 218,553 | 64.9 | 2973 |
| AKL12 | Ga0000124 | GCA_000377925.1 | Kulunda Steppe, Altai, Russia | 2,974,796 | 32 | 195,763 | 66.2 | 2833 |
| AKL17 | Ga0000125 | GCA_000377885.1 | Kulunda Steppe, Altai, Russia | 2,803,222 | 18 | 446,949 | 67.56 | 2700 |
| AKL19 | Ga0001414 | GCA_000420165.1 | Kulunda Steppe, Altai, Russia | 2,884,909 | 18 | 323,374 | 66.8 | 2752 |
| ALR17-21 | Ga0001423 | GCA_000420185.1 | Kulunda Steppe, Altai, Russia | 2,998,957 | 65 | 97,686 | 67.44 | 2918 |
| AL2^T^  (*Tv. versutus*) | Ga0073318 | GCA_001999325.1 | Transbaikal, Russia | 2,835,344 | 58 | 101,375 | 66.35 | 2702 |
| AL5 | Ga0000132 | GCA_000378565.1 | Buriatia, Russia | 2,980,438 | 11 | 607,277 | 65.67 | 2851 |
| AL21 | Ga0000131 | GCA_000381325.1 | Kenya, Africa | 2,966,254 | 5 | 1,077,549 | 65.36 | 2924 |
| ALD1 | Ga0000133 | GCA_000381245.1 | Buriatia, Russia | 2,644,105 | 12 | 567,188 | 65.19 | 2569 |
| ALE6 | Ga0001416 | GCA_000364565.1 | Wadi Natrun, Egypt | 2,963,704 | 61 | 97,183 | 66.76 | 2924 |
| ALE9 | Ga0000148 | GCA_000377445.1 | Wadi Natrun, Egypt | 2,938,401 | 24 | 223,577 | 67.09 | 2835 |
| ALE10 | Ga0001417 | GCA_000381385.1 | Wadi Natrun, Egypt | 2,766,032 | 21 | 348,567 | 67.7 | 2675 |
| ALE11 | Ga0001418 | GCA_000381205.1 | Wadi Natrun, Egypt | 2,985,038 | 55 | 215,431 | 67.31 | 2864 |
| ALE12 | Ga0000134 | GCA_000381105.1 | Wadi Natrun, Egypt | 2,850,306 | 24 | 304,386 | 67.28 | 2788 |
| ALE14 | Ga0000135 | GCA_000376845.1 | Wadi Natrun, Egypt | 3,024,612 | 31 | 215,672 | 66.6 | 2955 |
| ALE16 | Ga0000137 | GCA_000381305.1 | Wadi Natrun, Egypt | 3,141,511 | 39 | 148,095 | 66.61 | 3123 |
| ALE17 | Ga0000138 | GCA_000425605.1 | Wadi Natrun, Egypt | 2,776,528 | 27 | 277,261 | 67.26 | 2658 |
| ALE18 | Ga0000139 | GCA_000381465.1 | Wadi Natrun, Egypt | 2,767,238 | 23 | 348,709 | 67.71 | 2676 |
| ALE19 | Ga0001419 | GCF_000428605.1 | Wadi Natrun, Egypt | 3,118,282 | 67 | 97,026 | 67.1 | 3070 |
| ALE20 | Ga0000140 | GCA_000381405.1 | Wadi Natrun, Egypt | 2,845,558 | 28 | 322,207 | 67.59 | 2735 |
| ALE21 | Ga0000149 | NA | Wadi Natrun, Egypt | 2,853,770 | 23 | 212,617 | 67.12 | 2759 |
| ALE22 | Ga0000099 | GCA_000381445.1 | Wadi Natrun, Egypt | 2,748,237 | 20 | 260,302 | 67.33 | 2658 |
| ALE23 | Ga0001420 | GCA_000378545.1 | Wadi Natrun, Egypt | 2,896,574 | 66 | 449,267 | 67.33 | 2828 |
| ALE25 | Ga0000142 | GCA_000377285.1 | Wadi Natrun, Egypt | 2,855,242 | 22 | 219,607 | 67.11 | 2757 |
| ALE27 | Ga0000143 | GCA_000377485.1 | Wadi Natrun, Egypt | 2,745,684 | 22 | 146,879 | 67.36 | 2665 |
| ALE28 | Ga0000144 | GCA_000377425.1 | Wadi Natrun, Egypt | 2,910,020 | 29 | 188,636 | 67.12 | 2812 |
| ALE30 | Ga0000146 | GCA_000377465.1 | Wadi Natrun, Egypt | 2,779,335 | 17 | 272,801 | 67.35 | 2672 |
| ALE31 | Ga0000147 | GCA_000377405.1 | Wadi Natrun, Egypt | 2,770,361 | 16 | 411,808 | 67.33 | 2664 |
| ALEN2^T^  (*Tv. nitratireducens*) | Ga0012259 | GCA_000321415.1 | Wadi Natrun, Egypt | 4,002,352 | 1 | / | 66.5 | 3875 |
| ALgr1 | Ga0000106 | GCA_000378285. | Wadi Natrun, Egypt | 2,748,836 | 9 | 708,859 | 66 | 2641 |
| ALgr3 | Ga0000107 | GCA_000377325.1 | Wadi Natrun, Egypt | 2,881,569 | 61 | 246,902 | 67.6 | 2817 |
| ALgr5 | Ga0000141 | GCA_000381485.1 | Kulunda Steppe, Altai, Russia | 2,853,770 | 23 | 148,251 | 67.56 | 2726 |
| ALJ1 | Ga0000100 | GCA_000745395.1 | Kenya, Africa | 2,766,100 | 5 | 2,249,666 | 64.11 | 2656 |
| ALJ2 | Ga0000111 | GCA_000378325.1 | Kenya, Africa | 2,881,727 | 12 | 425,729 | 65.79 | 2754 |
| ALJ3 | Ga0000115 | GCA_000377205.1 | Kenya, Africa | 2,629,588 | 12 | 484,658 | 65.3 | 2532 |
| ALJ4 | Ga0000116 | GCA_000377225.1 | Kenya, Africa | 2,982,358 | 8 | 728,365 | 65.65 | 2857 |
| ALJ5 | Ga0000117 | GCA_000377245.1 | Kenya, Africa | 2,983,025 | 10 | 607,289 | 65.65 | 2853 |
| ALJ6 | Ga0000118 | GCA_000377365.1 | Kenya, Africa | 2,845,094 | 17 | 246,018 | 64.27 | 2723 |
| ALJ7 | Ga0000119 | GCA_000376865.1 | Kenya, Africa | 2,712,147 | 12 | 500,151 | 64.51 | 2617 |
| ALJ8 | Ga0000120 | GCA_000377385.1 | Kenya, Africa | 2,817,953 | 7 | 1,620,505 | 65.98 | 2693 |
| ALJ9 | Ga0000121 | GCA_000380585.1 | Kenya, Africa | 2,933,045 | 11 | 607,192 | 65.74 | 2803 |
| ALJ10 | Ga0000101 | GCA_000377305.1 | Kenya, Africa | 2,644,363 | 14 | 273,077 | 65.2 | 2566 |
| ALJ11 | Ga0000102 | GCA_000376925.1 | Kenya, Africa | 2,776,911 | 55 | 85,244 | 66.02 | 2643 |
| ALJ12^T^  (*Tv. nitratis*) | Ga0000108 | GCA_000378305.1 | Kenya, Africa | 2,779,390 | 6 | 1,574,912 | 65.99 | 2641 |
| ALJ15 | Ga0001412 | GCA_000383695.1 | Kenya, Africa | 2,852,324 | 13 | 547,002 | 65.88 | 2738 |
| ALJ16 | Ga0000109 | GCA_000377345.1 | Kenya, Africa | 2,755,057 | 32 | 165,740 | 66.98 | 2667 |
| ALJ17 | Ga0000103 | GCA_000377945.1 | Kenya, Africa | 3,053,023 | 69 | 74,794 | 65.15 | 2974 |
| ALJ20 | Ga0000112 | GCA_000378585.1 | Kenya, Africa | 2,965,657 | 5 | 1,060,159 | 65.37 | 2922 |
| ALJ21 | Ga0000113 | GCA_000378605.1 | Kenya, Africa | 2,964,639 | 5 | 1,077,390 | 65.38 | 2922 |
| ALJ24 | Ga0000151 | GCA_000377785.1 | Kenya, Africa | 2,814,898 | 20 | 318,966 | 67.48 | 2707 |
| ALJD^T^  (*Tv. denitrificans*) | Ga0073317 | GCA_002000365.1 | Kenya, Africa | 3,639,353 | 167 | 44,353 | 64.53 | 3486 |
| ALJT | Ga0000153 | GCA_000381825.1 | Kenya, Africa | 2,659,308 | 21 | 224,258 | 65.76 | 2523 |
| ALM2^T^  (*Tv. jannaschii*) | Ga0000154 | GCA_000381505.1 | Mono Lake, California, USA | 2,924,114 | 26 | 206,838 | 65.94 | 2800 |
| ALMg11 | Ga0000155 | GCA_000377905.1 | North-eastern Mongolia | 3,037,788 | 18 | 486,232 | 65.97 | 2947 |
| ALMg13.2 | Ga0000157 | GCA_000381185.1 | North-eastern Mongolia | 3,018,254 | 64 | 118,769 | 65.91 | 2938 |
| ALMg2 | Ga0000158 | GCA_000381145.1 | North-eastern Mongolia | 2,918,561 | 54 | 112,534 | 65.99 | 2836 |
| ALMg3 | Ga0000159 | GCA_000381225.1 | North-eastern Mongolia | 2,799,910 | 22 | 189,696 | 66.45 | 2671 |
| ALMg9 | Ga0001415 | GCA_000380625.1 | North-eastern Mongolia | 2,925,715 | 9 | 802,745 | 66.41 | 2819 |
| ALRh | Ga0001413 | GCA_000381425.1 | Kenya, Africa | 2,747,676 | 14 | 1,622,820 | 65.99 | 2635 |
| ALSr1 | Ga0000161 | GCA_000381945.1 | Searles Lake, California, USA | 2,928,811 | 25 | 301,221 | 67.46 | 2815 |
| ARhD1^T^  (*Tv. thiocyanodenitrificans*) | Ga0025308 | GCA_000378965.1 | Wadi Natrun, Egypt | 3,746,647 | 3 | 3,405,355 | 64.83 | 3679 |
| ARh1^T^  (*Tv. paradoxus*) | Ga0025151 | GCA_000227685.3 | Kenya, Africa | 3,756,729 | 1 | / | 66.59 | 3233 |
| ARh2^T^  (*Tv. thiocyanoxidans*) | Ga0001424 | GCA_000385215.1 | Kenya, Africa | 2,765,337 | 61 | 76,349 | 66.18 | 2677 |
| ARh3 | Ga0000104 | GCA_000377265.1 | Kenya, Africa | 2,788,198 | 15 | 447,462 | 66.17 | 2716 |
| ARh4 | Ga0000105 | GCA_000378265.1 | Buriatia, Russia | 3,043,512 | 10 | 609,229 | 65.61 | 2924 |
| ARh5 | Ga0000162 | GCA_000381805.1 | Wadi Natrun, Egypt | 2,768,380 | 8 | 679,738 | 66.16 | 2676 |
| HL-Eb18 | Ga0001422 | GCA_000364985.1 | Full-scale Thiopaq bioreactors | 2,761,862 | 35 | 149,474 | 65.81 | 2724 |
| HL-EbGr7^T^  (*Tv. sulfidiphilus*) | Ga0031212 | GCA_000021985.1 | Full-scale bioreactor removing sulfide | 3,464,554 | 1 | / | 65.06 | 3366 |
| HL17^T^  (*Tv. halophilus*) | Ga0073316 | GCA_001995255.1 | Kulunda Steppe, Altai, Russia | 2,871,788 | 97 | 70,770 | 67.5 | 2792 |
| K90mix | Ga0031213 | GCA_000025545.1 | Kulunda Steppe, Altai, Russia | 2,985,056 | 1 | / | 65.54 | 2942 |
