# Supplementary material for: Genomic diversity within the haloalkaliphilic genus Thioalkalivibrio
Source: PLoS One. 2017 Mar 10;12(3):e0173517. doi: 10.1371/journal.pone.0173517 (PMC5345834; doi:10.1371/journal.pone.0173517)
Supplement: S2 Table — (DOCX) [file pone.0173517.s003.docx]

S2 Table. Genome characteristics of the other genera used in this study.

| **Strain name** | **GOLD ID** | **Genbank assembly accession** | **Location of isolation** | **Genome size (Mbp)** | **No. of scarfolds** | **Scaffold N50** | **G/C ratio (%)** | **Predicted CDS** |
| --- | --- | --- | --- | --- | --- | --- | --- | --- |
| A12^T^  (*Thiorhodospira siberica*) | Ga0024988 | GCA_000227725.2 | Lake Malyi Kasytui, Siberia, Russia | 3,188,869 | 186 | 47,937 | 56.63 | 2990 |
| ATCC 51935^T^  (*Ectothiorhodospira haloalkaliphila*) | Ga0025088 | GCA_000633935.1 | Wadi Natrun, Egypt | 3,448,426 | 4 | 1,813,775 | 62.99 | 3282 |
| MLHE-1^T^  (*Alkalilimnicola ehrlichii*) | Ga0027409 | GCA_000014785.1 | Mono Lake, California, USA | 3,275,944 | 1 | / | 67.53 | 2952 |
| SL1^T^  (*Halorhodospira halophila*) | Ga0028991 | GCA_000015585.1 | Summer Lake, Lake County, OR | 2,678,452 | 1 | / | 67.98 | 2470 |
| HL3^T^  (*Thiohalospira halophila*) | Ga0052857 | NA | S-W Siberia, Russia | 2,687,697 | 23 | 248,128 | 69.12 | 2703 |
